# Converging lines of evidence fail to detect nanovirus replication in its insect vector

**DOI:** 10.1099/jgv.0.002354

**Published:** 2026-10-27

**Authors:** Laura Di Pietro, Mathilde Villegas, Auguste Rajot, Nina Lukhovitskaya, Valentin Guyot, François Hoh, Yasmine Tiberbache, Jean-Louis Zeddam, Yannis Michalakis, Mikhail M. Pooggin, Stéphane Blanc

**Affiliations:** 1 PHIM, INRAE, CIRAD, IRD, Institut Agro, Univ Montpellier, Montpellier, France; 2 CBS, INSERM, CNRS, Univ Montpellier, Montpellier, France; 3 MIVEGEC, CNRS, IRD, Univ Montpellier, Montpellier, France

**Keywords:** Arbovirus, plant virus, vector transmission, aphid, nanovirus, circulative transmission, multipartite virus

## Abstract

Nanoviruses have a multipartite circular ssDNA genome and are transmitted by aphids in a circulative manner. Although generally considered non-propagative within their vectors, direct experimental evidence supporting the absence of viral replication remains limited. This question is particularly relevant for multipartite viruses because replication within vectors could theoretically help maintain genome integrity during transmission, decreasing dilution effects and the probability of genome segment loss. Here, we investigated whether faba bean necrotic stunt virus (FBNSV, genus *Nanovirus*, family *Nanoviridae*) replicates in aphids using several independent approaches. Viral load dynamics were quantitative PCR-monitored after virus acquisition, the replication-associated protein M-Rep was searched for within gut and salivary gland cells by immunofluorescence, viral DNA localization and strand specificity were analysed using HCR-DNA-FISH (hybridization chain reaction fluorescence *in situ* hybridization) and confocal microscopy, viral transcription was investigated by Illumina mRNA sequencing, and viral DNA replication was analysed by Nanopore sequencing. All approaches converged towards the same conclusion. Viral loads did not increase after acquisition, M-Rep was undetectable in aphid tissues, viral DNA accumulated outside nuclei, no negative-strand viral DNA was detected, and only negligible quantities of viral transcripts and dsDNA molecules were detected in RNA-seq and Nanopore-seq datasets from viruliferous aphids, although these molecules were readily detected in FBNSV-infected plants used as the acquisition source. While none of these approaches could alone suffice to definitely exclude a weak, localized and/or transient replication, they together strongly support the conclusion that FBNSV is transmitted in a genuinely circulative non-propagative manner.

## Data Availability

All data necessary for conclusions are presented in the manuscript. Raw qPCR data are provided as supplementary material as well as sequences of probes for HCR-DNA-FISH and detailed scripts of the statistical analysis. Raw data of the Illumina mRNA-seq and Nanopore DNA-seq analyses were deposited in the NCBI SRA database under the BioProject PRJNA1510737 including six insect mRNA-seq datasets ALYU-398-403 (biosamples SAMN62345578-SAMN62345583), six plant mRNA-seq datasets ALYU-416-421 (biosamples SAMN62317259-SAMN62317264), one insect Flongle DNA-seq dataset (biosample SAMN62696347) and one plant Flongle DNA-seq dataset (biosample SAMN62607744).

## Introduction

 The family *Nanoviridae* comprises plant-infecting circular ssDNA viruses that remain far less mechanistically characterized than those of the related family *Geminiviridae* [1]. This is most likely because of the tremendous agricultural importance of numerous geminivirus species on many distinct crops and all around the world [2], while nanoviruses are thus far less economically damaging, except for a few species of the genus *Nanovirus* on legumes and of the genus *Babuvirus* on banana, ginger or cardamom [3]. Consequently, several key aspects of their biology are still inferred from what has been established in geminiviruses rather than directly demonstrated in nanoviruses [1, 3–6]. This is particularly true for their mode of transmission by aphid vectors [5]. Nanoviruses are generally considered to be transmitted in a circulative non-propagative manner: virus particles are acquired from infected plants, cross vector midgut epithelial barriers, reach the salivary glands and are eventually inoculated into new host plants without replicating within the insect cells. Although the circulative nature of this process is well supported, the non-propagative component has mostly remained an assumption.

This assumption is likely inherited from the following context. In geminiviruses, the question of within-vector replication has been debated for decades, especially in begomoviruses transmitted by whiteflies [7–10]. The current view is that replication in the vector may occur in a very limited number of virus species, tomato yellow leaf curl virus (TYLCV) and perhaps a few closely related species [5]. Most other begomoviruses and apparently all other geminivirus genera do not replicate in their respective vectors [5]. By extension, nanoviruses have been placed in the same conceptual box: circular ssDNA plant viruses, transmitted by phloem-feeding insects but not replicating in them [3]. Yet, when looking for empirical evidence in nanoviruses, the basis is surprisingly thin. To our knowledge, the direct experimental support comes from a single three decades-old study on the banana bunchy top virus (BBTV, genus *Babuvirus*), in which Southern blot hybridization analysis of BBTV-infected banana plants revealed monomeric, dimeric and other multimeric double-stranded forms of viral DNA, interpreted as evidence for rolling-circle replication, whereas only monomeric viral ssDNA was detectable in viruliferous aphids [11]. Beyond this, the family *Nanoviridae* has essentially remained under a ‘non-propagative by default’ status.

The question nevertheless deserves direct attention. Nanoviruses are multipartite viruses with six to eight circular genome segments, each individually encapsidated [12]. This genome organization and packaging together with the high number of segments raises a conceptual problem: how can such viruses maintain genome integrity along the various phases of their life cycle? [13]. In this context, replication within the vector could intuitively appear as one possible ‘re-assembling’ mechanism, allowing viral segments acquired at different frequencies [14] and eventually from distinct infected host plants [15] to be co-amplified before inoculation into a new plant. While speculative, this idea illustrates why investigating replication in the vector may not be a trivial detail. It is also fundamentally important because a strictly non-propagative circulative transmission appears rare and likely restricted to plant viruses, as it has never been reported for arboviruses infecting animals [16].

Our recent observations on the faba bean necrotic stunt virus (FBNSV) further reopened this question. The genome formula of FBNSV, i.e. the relative frequency of each genomic segment within the viral population [17], changes during the passage through aphid vectors [18]. At first sight, such changes could be compatible with differential replication of viral segments within the insect. However, subsequent work suggested that differential degradation could also explain these shifts without invoking replication [19]. This ambiguity motivated the present study where we did not aim to prove the absence of replication in a strict logical sense. Proving the absence of a biological process, by essence, is too often incomplete and thus questionable. Instead, we asked whether any detectable sign of replication could be found using a broad set of independent approaches targeting different hallmarks of viral multiplication within the vector. If nanoviruses replicate in aphids, one may expect to observe at least some of the following: an increase in viral load after acquisition, detection of the replication-associated M-Rep protein in vector tissues, localization of viral DNA in nuclei where replication of ssDNA virus occurs, detection of the complementary negative DNA strand present in circular double-stranded replicative intermediates, production of viral mRNAs and formation of long dsDNA molecules (as previously observed in BBTV-infected banana plants).

Here, we combine quantitative PCR (qPCR), immunolocalization, strand-specific DNA-HCR-FISH (hybridization chain reaction fluorescence *in situ* hybridization), confocal imaging, Illumina sequencing of viral mRNA and Nanopore sequencing of viral dsDNA to assess whether FBNSV replicates in its aphid vector. These approaches differ in principles, sensitivities and limitations, but they all converge toward the same conclusion: we failed to detect any convincing evidence for replication of FBNSV in aphids. Our results therefore strongly reinforce the view that FBNSV, and perhaps other nanoviruses, are transmitted by their aphid vectors in a truly non-propagative manner.

## Methods

### Plants, aphids and virus isolate

Faba bean plants (*Vicia faba ‘Seville’, La Bonne Graine, France*) were used as the main host plant throughout this study. Plants were seeded and maintained in insect-proof growth chambers programmed for a 13/11 h light/dark cycle with day/night temperatures of 25/18 °C and a constant relative humidity of 60%.

Colonies of *Acyrthosiphon pisum* (clone LL01) were reared on uninfected faba bean plants under similarly controlled environmental conditions. Aphid colonies were regularly transferred onto non-infected plants to maintain virus-free parthenogenic lines and cohorts. Acquisition of the virus by aphids was allowed during an acquisition access period (AAP) that is specifically indicated for each experiment in the next section of the Methods.

The FBNSV isolate JKI-2000 was used throughout this study. The infectious clone has been earlier described [20]. In brief, each of the eight genome segments named C, M, N, R, S, U1, U2 and U4 has been inserted as a tandem repeat into the binary plasmid pBin19 and transformed in *Agrobacterium tumefaciens* strain COR308.

### Agroinoculation, production of infected plants and virus acquisition by aphids

The eight agrobacteria, each transformed with a distinct FBNSV genome segment, were cultured and equal volumes were mixed prior to agro-inoculation, as earlier described [15]. The first developing leaves of young plants’ shoot were punctured with a needle and then agro-infiltrated using a needleless syringe on the abaxial side. Plants were maintained in growth chambers and infection was monitored for symptom development and qPCR detection of viral DNA 21 days post-inoculation.

Synchronized aphids were allowed to feed on apical leaves of infected plants or uninfected controls for defined AAPs, the duration of which depended on the experiment. For qPCR monitoring of the viral load, the AAP lasted 8 h, whereas it lasted 96 h for total RNA/DNA extraction preceding Illumina mRNA sequencing and Nanopore dsDNA sequencing. For confocal microscopy observation after immunofluorescence and HCR-DNA-FISH, aphids were allowed an AAP of 10 days in order to maximize accumulation and thus fluorescent signals, as earlier reported [21]. Importantly, the 4- and 10-day acquisition periods largely exceed the time required for FBNSV to complete the vector-internal steps leading to successful transmission, thereby ensuring that sufficient time had elapsed for any replication event potentially associated with transmission.

### DNA extraction and quantitative PCR

After storage at −80 °C, individual aphids were homogenized in the lysis buffer provided with the PureLink™ Plant Total DNA Purification Kit (Invitrogen). Total DNA was extracted from whole aphids according to the manufacturer’s protocol and eluted in 50 µl of nuclease-free water. Viral DNA was quantified by qPCR using undiluted DNA extracts. Amplifications were performed on a LightCycler^®^ 480 Instrument II (Roche) following the protocol earlier described by [21]. Each reaction was carried out using the FastStart DNA Master Plus SYBR Green I kit (Roche) according to the manufacturer’s recommendations. The thermal cycling programme consisted of an initial denaturation step followed by 40 cycles of 95 °C for 10 s, 60 °C for 10 s and 72 °C for 10 s. Viral genomic segments were detected using the primer pairs described by [17]. Raw fluorescence data were analysed using the LinRegPCR software and viral DNA concentrations were subsequently determined using standard curves, following the approach described previously [22]. Differences in viral load among sampling time points and between plant samples were evaluated using the Scheirer–Ray–Hare test, a non-parametric ANOVA equivalent that does not require normality of residuals or homogeneity of variances. All statistical analyses were performed in R, and detailed results are provided in the Supplementary Materials.

### Immunofluorescence detection of M-Rep

Anterior midguts (AMGs) and salivary glands were dissected from viruliferous and non-viruliferous adult aphids in 1× PBS and subsequently fixed in 4% paraformaldehyde (PFA) for 20 min. Fixed plant stems (4% PFA overnight) were embedded and then sectioned (100 µm), according to the protocol earlier described [23]. Then, plant sections were cleared in ClearSee solution (10% wt/v xylitol, 15% wt/v sodium deoxycholate and 25% wt/v urea) for 48 h at room temperature (RT) as described previously [23]. To facilitate antibody penetration, aphid samples were treated with proteinase K (1 µg ml^−1^) for 10 min at RT and transferred in PBST-glycine (1× PBS containing 0.05% Tween-20 and 0.1 M glycine) for 10 min. Samples were then rinsed three times for 5 min with PBST (1× PBS containing 0.05% Tween-20) and fixed 15 min in 4% PFA. Plant samples were not treated with proteinase K because sectioning sufficiently facilitates the entry of antibodies into the intracellular compartment. All samples were then rinsed three times 5 min in PBST and incubated for 1 h 30 min in 5% BSA solution in PBST. They were finally transferred in the primary antibody solution (in PBST, dilution 1/200) overnight at 4 °C. The next day, they were rinsed three times 5 min in PBST at RT and incubated for 1 h at 37 °C with the secondary antibodies mixture at 1/200. Samples were then rinsed three times 5 min with PBST and maintained in 1× PBS until further processing.

For coat protein (CP) detection, a mixture of mouse monoclonal antibodies CP 8–2 G10 and CP 8–6 F8 [24] were used. M-Rep was detected using a custom rat polyclonal antibody raised against the peptide sequence CKEESRIEGPWEFGEFIAS (Eurogenetec) and secondary antibodies consisted of the Alexa Fluor 488-conjugated goat anti-mouse IgG and the Alexa Fluor 594-conjugated goat anti-rat.

### HCR-DNA-FISH for specific detection of plus and minus viral strand

Localization of FBNSV DNA within aphid and plant tissues was performed using HCR-FISH. Upper stems and petioles of infected *V. faba* plants were fixed, embedded and sectioned according to the protocol earlier described [23]. Dissected aphid organs were fixed as described above for immunofluorescence with a fixation time extended to 40 min. Plant cross-sections clearing was performed using ClearSee solution for 48 h at RT, as described [25]. After clearing and washing with PBS in case of plant cross-sections, or directly after fixation in the case of aphid organs, samples were subjected to HCR-FISH hybridization according to [26], with one modification: a denaturation step was added to allow detection of dsDNA. After prehybridization and addition of the hybridization buffer containing the probe mixture, samples were incubated at 85 °C for 4 min and then immediately transferred to a 37 °C hybridization oven.

The coding sequence of the FBNSV U4 segment was used as a target for the detection of dsDNA, ssDNA and mRNA, while its antisense sequence was used to detect negative-sense DNA within the replicative form. Four pairs of probes were designed for each target using HCR Probe 3.0 Maker (https://github.com/rwnull/insitu_probe_generator) [27, 28]. The probes and amplifiers used are listed in Table S1, available in the online Supplementary Material.

### Microscopy observation

Dissected aphid organs or plant sections treated for HCR-DNA-FISH or immunolocalization were mounted in Vectashield^®^ PLUS (antifade mounting medium with DAPI on microscopy slides). Observations and image acquisition were carried out with a Zeiss LSM 900 inverted confocal microscope (Carl Zeiss, Oberkochen, Germany) with 20× and/or 63× objectives. Images correspond either to maximum intensity projections of confocal z-stacks or to single z-planes, as indicated. Depending on the experiment, excitation was performed using 488, 561 and/or 640 nm laser lines. The setting of parameters for image acquisition was adjusted to optimize signal detection depending on the tissue observed but were kept identical within each comparison (viruliferous vs non-viruliferous or uninfected vs infected).

### RNA extraction and Illumina mRNA sequencing analysis of plants and insects

Total RNA was extracted from leaf tissue samples of *V. faba* plants infected with FBNSV and from control mock-inoculated plants (three plants for each of the two conditions), 15 days after aphid-mediated inoculation and 4-day infestation with virus-free *A. pisum* aphids. Total RNA was also extracted from pooled samples of the *A. pisum* aphids used for the 4-day AAP and collected from the respective plants (one pool per plant, three pools for each of the two conditions). A Cetyl Trimethyl Ammonium Bromide (CTAB) protocol [29] was used for plant RNA extraction, while the Trireagent^®^ protocol from Genbiotech was used for insect RNA extraction. RNA quantity, purity and integrity were verified using Nanodrop spectrophotometry and capillary electrophoresis on LabChip GX (Perkin Elmer). Illumina sequencing was performed at Fasteris SA (https://www.fasteris.com/) using the total RNA extracts for library preparation with the Illumina TruSeq Stranded mRNA protocol: poly-A selection, transcript fragmentation, first-strand cDNA synthesis with random primers, second-strand cDNA synthesis, ligation of indexed adapters, amplification of the library, followed by quantitation and 100-nt paired-end sequencing of the plant and insect cDNA libraries in two separate SP flowcells of NovaSeq 6000 (one for plants and one for aphids) to avoid cross-contamination through index swapping. The generated output from the six plant libraries ranged from 47 to 51 million reads, with Q30 ranging from 92.4 to 93.8, while that from the six insect libraries ranged from 46 to 51 million reads, with Q30 ranging from 92.5 to 93.9. After demultiplexing, reads from each library were mapped with Burrows–Wheeler Aligner 0.7.12 to the reference sequences of the eight segments of the viral genome, and the viral genome-mapped reads were combined for each condition.

### DNA extraction and nanopore sequencing

Total DNA was extracted from a FBNSV-infected *V. faba* plant using a Matab protocol and from a pool of non-purged *A. pisum* aphids born on a FBNSV-infected *V. faba* plant using a TNES protocol (both described in [30]). For sequencing on Oxford Nanopore MinION Mk1B device, libraries were prepared using a ligation sequencing kit (SQK-LSK109) according to the manufacturer’s protocol with some modifications. Briefly, ~3 µg of total DNA in a volume of 48 µl were mixed with 3.5 µl NEBNext FFPE DNA Repair Buffer, 2 µl NEBNext FFPE Repair mix, 3.5 µl Ultra II End-prep reaction buffer and 3.5 µl Ultra II End-prep enzyme mix, followed by incubation at 20 °C for 15 min, heating at 65 °C for 5 min and then cooling at RT. The DNA repair reaction of 60 µl was mixed with equal volume of resuspended AMPure XP beads and incubated on a Hula rotator mixer for 5 min at RT. The beads were washed twice with 200 µl of fresh 70% ethanol, dried for 30 s and resuspended in 70 µl nuclease-free water. The eluate DNA (60 µl) was mixed with 25 µl ligation buffer, 10 µl NEBNext Quick T4 DNA ligase and 5 µl adapter mix, followed by incubation for 20 min at RT. The ligation reaction of 100 µl was mixed with 40 µl resuspended AMPure XP beads and incubated on a Hula rotator mixer for 5 min at RT. The beads were washed twice with 250 µl long fragment buffer, dried for 30 s and then resuspended in 14 µl elution buffer and incubated for 15 min at 37 °C. For control of DNA integrity and quantity, 1 µl eluate was analysed on 0.8% agarose gel and 1 µl on Qubit fluorometer. Finally, the library was loaded on a Flongle R9.4.1 flowcell and sequenced for 42 h. The plant and aphid DNA libraries were sequenced in two separate flowcells, with a total output of 745,477,697 and 697,361,536 bases, respectively, after base-calling with Dorado (dna_r9.4.1_e8_hac@v3.3). The basecalled reads from each library were mapped using Minimap2 (v2.28 with the map-ont preset) to the reference sequences of the eight segments of the FBNSV genome, followed by calculation of the number of viral reads and the percentage of viral bases (the sum of bases in the viral reads) in the total number of sequenced (viral+host) bases in the plant and the aphid samples.

## Results

### The viral load in whole aphids does not increase after virus acquisition

We first addressed the possibility of FBNSV replication in aphids using the most direct and intuitive approach: monitoring viral accumulation over time after virus acquisition. Groups of 30 aphids were deposited on three infected source plants and allowed to feed for 8 h. They were then removed from their source plants and immediately transferred into 1.5 ml Eppendorf tubes (experiment 1) or into cylindrical copper chambers sealed with a parafilm membrane (experiment 2), through which they could feed into pure water in order to minimize dehydration. Viral DNA quantities were then measured in individual aphids immediately after removal from the plants (T0) and after 12 and 24 h. To properly account for elevated inter-individual variability, 21 aphids (seven per infected source plant) were analysed individually for each time point. Fig. 1a and b show the distribution of data in the two experiments. Fig. 1 shows no increase in viral load with time after removal from infected plants (Scheirer–Ray–Hare test, time factor *P*-value=0.719 for experiment 1; and Scheirer-Ray-Hare test, time factor *P*-value=0.163 for experiment 2). Both raw quantification data of the viral load (Tables S2 and S3), as well as full statistical analyses are provided in the Supplementary Material.

**Fig. 1.**
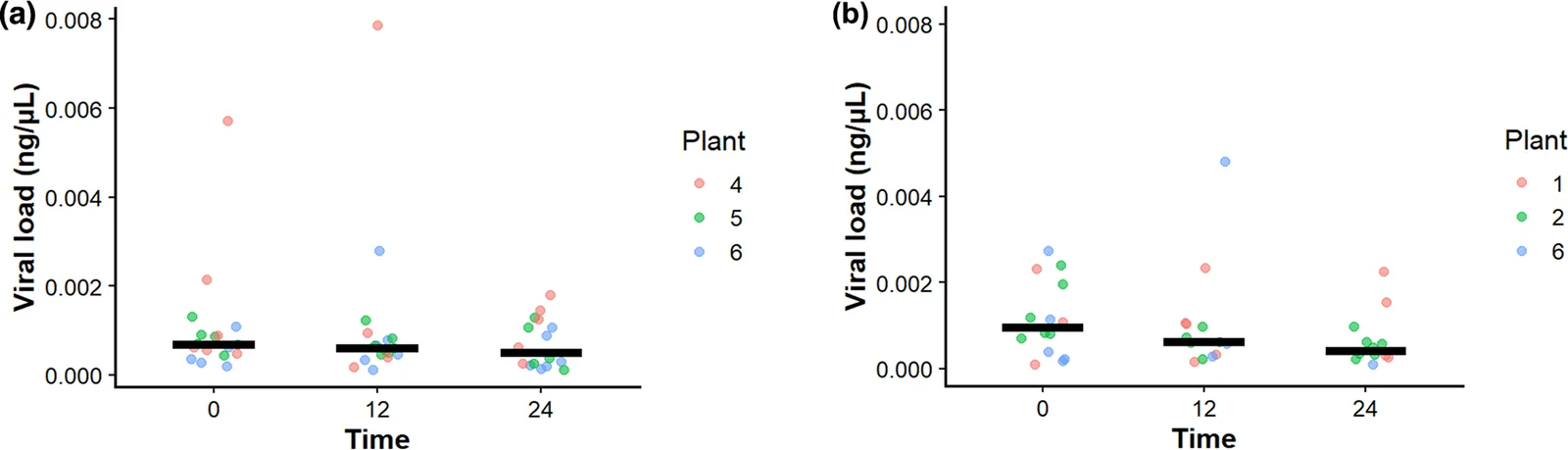
Total viral load in viruliferous *A. pisum* after removal from the infected plants. Non-viruliferous aphid individuals were placed on three different FBNSV-infected faba bean source plants for 8 h and then removed from these plants (T0). The viral load was quantified by qPCR as described in the Methods on 21 individual aphids (seven from each of the three infected source plants) at T0, 12 h (T12) and 24 h (T24) post-removal. (a) and (b) respectively represent the two experiments described in the text. Only individual aphids showing positive detection of FBNSV were maintained in the analysis. In (a), 17 aphids tested positive at T0, 17 at T12 and 16 at T24. In (b), 14 aphids tested positive at T0, 13 at T12 and 12 at T24. For each time point, the distribution of viral load values is directly reported as one dot per individual aphid, where each colour indicates a different source plant. The horizontal bar shows the median value for all aphids in one time point. Details of the statistical test are provided in Supplementary statistical analyses.

### No M-Rep protein could be detected in aphid cells

Replication of circular ssDNA viruses critically depends on the expression of their replication-associated protein (Rep) [31]. In nanoviruses, the master replication protein (M-Rep) is indispensable for rolling-circle replication of viral DNA segments [32]. We therefore investigated whether this protein could be detected in aphid tissues known to accumulate FBNSV particles [21], the AMG and principal salivary glands (PSG). Immunofluorescence experiments using custom-produced M-Rep-directed IgGs (see Methods) were performed on dissected AMG and PSG from viruliferous aphids. Infected and uninfected plant tissues as well as non-viruliferous aphids were used as positive and negative controls. A strong and unambiguous M-Rep detection was readily obtained in infected plant cells but not in uninfected plants, validating antibody specificity and accessibility of the targeted epitopes (Fig. 2a1 and a2). In contrast, despite numerous individuals processed, no detectable M-Rep signal could be observed, neither in AMG (Fig. 2a3 and a4) nor in PSG (Fig. 2a5 and a6) cells, of both viruliferous and control non-viruliferous aphids.

**Fig. 2.**
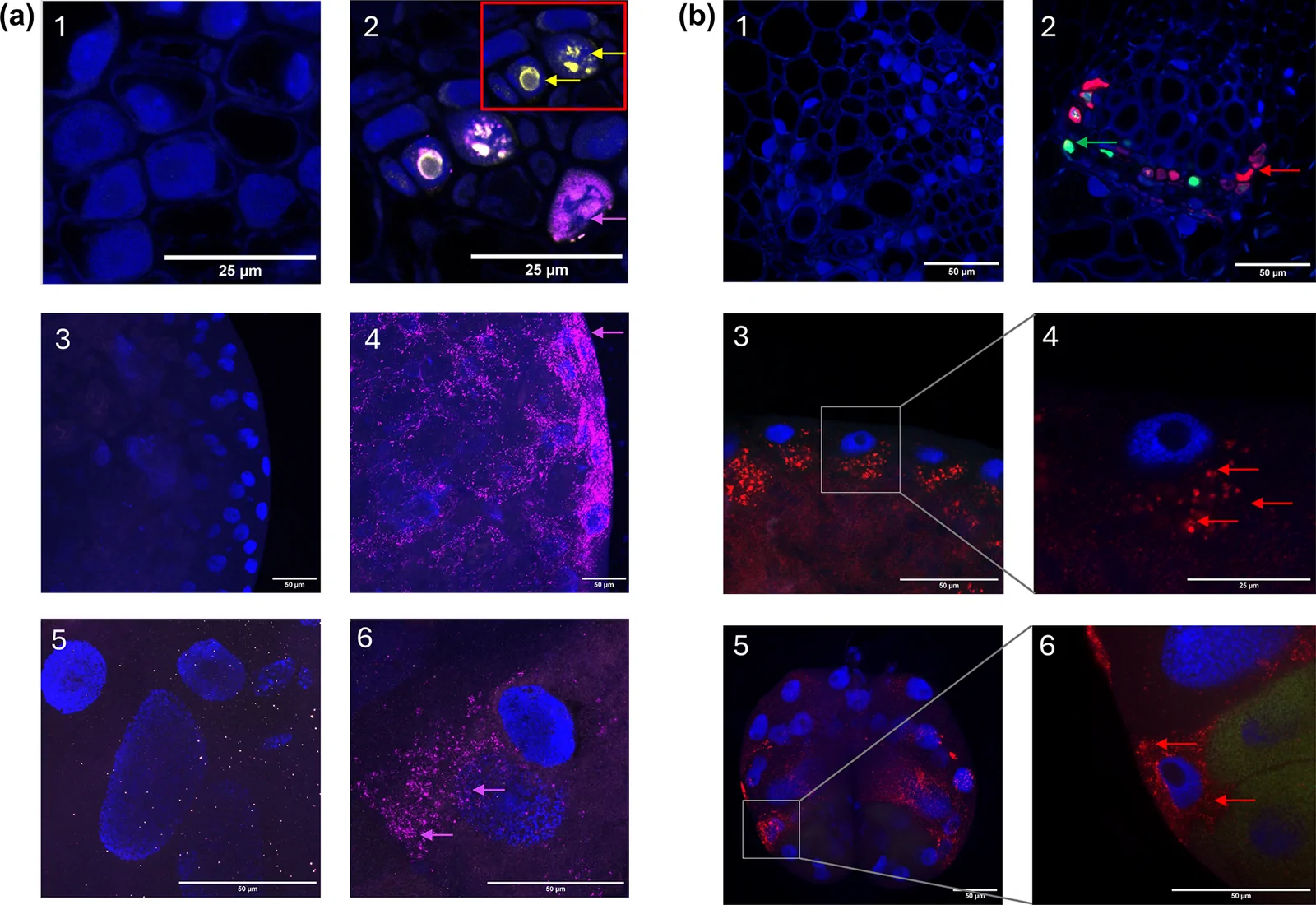
No FBNSV replication factor nor replication intermediate can be detected in aphid vector cells. (a) Immunofluorescence detection of the viral M-Rep (yellow) and CP (magenta) proteins in infected plants and aphids. A strong and specific M-Rep signal was observed in phloem cells of infected plants (stem sections, a2), whereas no signal was detected in uninfected plants (a1). The red inset in a2 is showing only the M-Rep detection channel to facilitate visualization of the M-Rep signal, which largely overlaps with the CP signal in the merged image. In aphids, M-Rep was undetectable in both AMG (a3–a4) and PSG (a5–a6) from either viruliferous (a4, a6) or non-viruliferous (a3, a5) individuals, despite a strong CP signal in viruliferous aphids, confirming viral accumulation and validating tissue integrity. Panels a3–a6 correspond to orthogonal projections of confocal Z-stacks. Scale bars are indicated in each panel. Nuclei were stained with DAPI (blue). Images shown are representative of observation from seven dissected midguts and salivary glands in which CP was successfully detected. (b) HCR-DNA-FISH detection of FBNSV (+)ssDNA (red) and (-)ssDNA (green) (U4 segment) in infected and uninfected tissues. Stem sections from uninfected (b1) and infected (b2) *V. faba* plants, as well as AMG cells (b3, X20; b4, X63) and PSG cells (b5, X20; b6, X63) from viruliferous aphids, were analysed. (+)ssDNA was detected in infected plant tissues and in aphid vector cells, displaying an exclusively cytoplasmic localization. In contrast, (-)ssDNA signal was detected in plant tissues but not in aphid samples. Nuclei were stained with DAPI (blue). Images correspond to a single confocal Z-plane, and scale bars are indicated in each panel. Images shown are representative of observations from ~10 non-viruliferous and 10 viruliferous aphids, and 2 independent plants.

### Viral DNA accumulates outside nuclei in aphid cells

Replication of gemini- and nanoviruses is expected to occur in the nucleus, where host DNA polymerases synthesize the complementary strand and generate circular double-stranded replicative intermediates [31, 33], and where the replication initiator protein M-Rep recruits host DNA polymerases [6, 34, 35]. We therefore re-examined the intracellular localization of FBNSV DNA in aphid tissues using HCR-DNA-FISH coupled with confocal microscopy. Analysis of confocal single z-plane images confirmed abundant viral DNA accumulation in both AMG and PSG cells (Figs. 2b3-b4 and b5-b6, respectively). However, despite such a high accumulation, these images consistently showed that viral DNA fluorescent foci were excluded from insect cell nuclei. Viral signals instead appeared restricted to the cytoplasmic compartment. These observations are difficult to reconcile with active replication of a circular ssDNA virus and instead support a transcytosis-like transport process through vector tissues, as classically described for circulative non-propagative viruses [36].

### No viral negative-strand DNA is detectable in aphid tissues

Because synthesis of the complementary strand is an obligatory step in the replication cycle of circular ssDNA viruses, to further investigate the possible existence of replicative forms in aphid cells, we developed a strand-specific adaptation of HCR-DNA-FISH allowing independent detection of positive and negative viral DNA. Probe sets were designed against segment U4, which is relatively abundant in within-host and vector viral populations [17, 18]. In infected plant tissues, both positive and negative strands were readily detected, producing strong fluorescent signals fully consistent with active synthesis of double-stranded replication intermediates. It is remarkable that the negative strand is relatively easy to detect in planta (Fig. 2b2), despite the fact that only the positive strand accumulates in the form of packaged genomic DNA. In viruliferous aphids (Fig. 2b3 to B6), by contrast, such replication intermediates could not be detected. Despite the very strong accumulation of positive-strand DNA in AMG and PSG cells, no detectable negative-strand signal was observed in any aphid tissue examined.

The absence of detectable negative-strand DNA provides additional evidence against active FBNSV replication within aphids.

### Viral mRNAs are virtually absent from RNAseq datasets of viruliferous aphids

Replication of nanoviruses requires prior transcription of viral genes, most notably those encoding the replication protein M-Rep. Thus, even in the hypothetical situation where replication itself remained difficult to detect, substantial viral replication activity would be expected to rely on detectable viral transcripts. To test this possibility, we performed Illumina mRNA sequencing analysis of aphids exposed for 4 days to FBNSV-infected plants. Three biological replicates were performed, and high sequencing depths were obtained maximizing sensitivity. Despite the hundreds of thousands of viral reads generated in the infected plant libraries, viral RNA reads detected in viruliferous aphids remained extremely rare (Fig. 3a). In fact, only 18 and 4 such reads were found in aphids of two out of three biological replicates, while no reads were detected in the third replicate. This observation, together with the 35 reads detected in one of the three biological replicates of mock-inoculated control plants, could be due to cross contamination between the sequenced samples. Alternatively, and actually expected, a few viral reads could result from viral mRNAs ingested by aphids from infected plants and still not fully degraded in the gut lumen at the time of extraction. In any case, the quasi-absence of viral reads in the transcriptome of viruliferous aphids is incompatible with a potentially functional viral transcriptional activity in aphid cells.

**Fig. 3.**
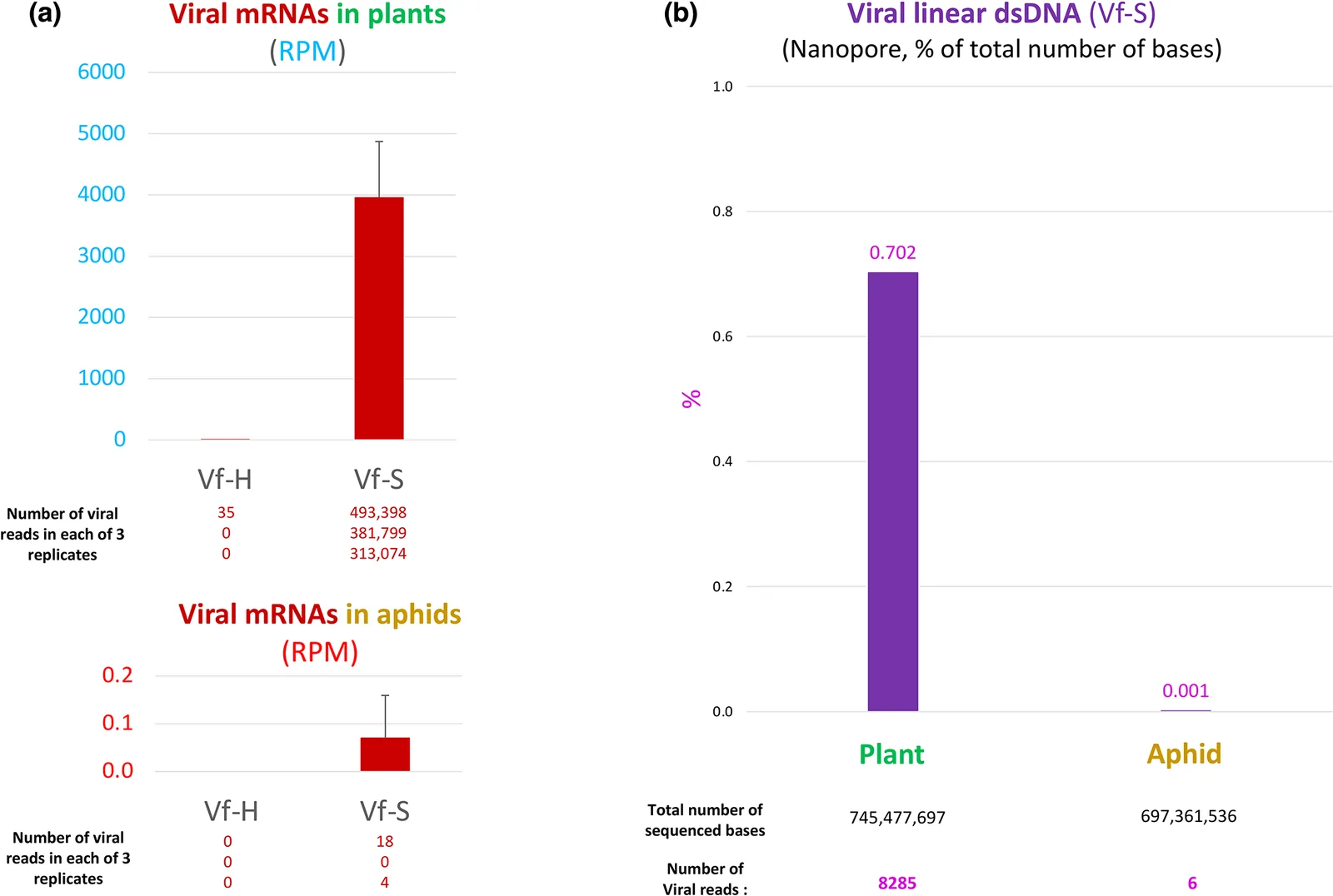
Search for viral transcription and replication products within viruliferous aphids. (a) Illumina-seq analysis of viral mRNA accumulation in mock-inoculated (Vf-H) and FBNSV (Vf-S)-infected *V. faba* plants and, in aphids fed for 4 days on these plants. Total RNA was extracted from plants and aphids, sequenced and analysed, as explained in the Methods section. The bar graphs show the accumulation of viral mRNAs normalized in reads per million of total (viral+host) mRNA reads in each of the two conditions for plant (upper graphs) and aphids (lower graphs). Error bars indicate the sd of the mean values. The number of non-normalized viral mRNA reads sequenced from each of the three biological replicates per condition are given below the bar graphs. (b) Nanopore-seq analysis of viral linear dsDNA accumulation in FBNSV (S)-infected *V. faba* plant (Vf) and viruliferous *A. pisum* aphids. Total DNA was extracted from leaf tissue and from aphids fed on it, Nanopore-sequenced, and analysed, as described in the Methods. The bar graph shows the percentage of viral bases (sum of bases in the viral reads) in the total number of sequenced bases (viral +host) in the plant and the aphid sample. For each sample, the total number of sequenced bases and the number of viral DNA reads are given below the bar graph.

### Negligible amount of viral dsDNA could be detected in aphids

Circular ssDNA viruses of the family *Geminiviridae* replicate via rolling circle replication generating multiple copies of circular ssDNA and recombination-dependent replication generating linear dsDNA molecules of heterogeneous length [37, 38]. Since long dsDNA molecules have also been detected in babuvirus BBTV-infected plants [11], we searched for such replication-associated dsDNA molecules using Nanopore sequencing of total DNA extracts from FBNSV-infected plants and viruliferous aphids.

In infected plant samples, Nanopore sequencing revealed abundant viral dsDNA (Fig. 3b). In striking contrast, only a few FBNSV-derived dsDNA molecules (*n*=6) could be identified in aphid samples, which constituted 0.001% of total (viral +aphid) dsDNA, compared to 8,285 molecules identified in planta, constituting 0.702% of total (viral +plant) dsDNA. This huge (ca. 700-times) difference between plants and aphids indicates an absence of viral replication in aphids. Just as for mRNA above, these few reads identified in aphids may stem from viral dsDNA potentially ingested from plants and not fully degraded within the aphid gut lumen.

## Discussion

Circulative non-propagative transmission is a peculiar mode of virus circulation through an insect vector [16]. In contrast to animal arboviruses, for which biological transmission is classically associated with replication in the vector, several plant viruses can cross insect tissues, reach the salivary glands and be inoculated into new hosts without any recognized multiplication step [36]. Yet, the strictly non-propagative nature of this process has not always been easy to establish. In plant ssDNA viruses, and especially in begomoviruses, the possible replication of viral genomes within vectors has been repeatedly debated, with different studies reaching apparently conflicting conclusions depending on the virus isolate, vector population, tissue examined, marker used and interpretation of weak or localized signals. These controversies have been discussed in detail recently [5]. This context is important because it illustrates a general methodological difficulty: a single observation is rarely sufficient to either demonstrate or confidently dismiss within-vector replication.

In the case of FBNSV, revisiting this question appeared particularly relevant because previous observations suggested that changes in genome formula during aphid transmission could, at least theoretically, be compatible with differential amplification of viral segments within the vector [18]. We therefore approached the question without assuming that any individual method would be decisive. Viral load kinetics in Fig. 1 provided a direct and intuitive test, but whole-insect measurements may miss rare replication events restricted to a small number of cells or masked by simultaneous degradation elsewhere, as it has apparently been the case for TYLCV [9]. By contrast, immunolocalization of M-Rep and HCR-DNA-FISH approaches directly target the specific aphid organs and cells involved in virus circulation and therefore offer increased spatial resolution and sensitivity for localized events [39]. Immunodetection of M-Rep tests whether this essential replication initiator accumulates in aphid cells, while intracellular localization and strand-specific HCR-DNA-FISH test whether viral DNA reaches the nuclear compartment expected for circular ssDNA virus replication and whether complementary negative-strand intermediates are produced. RNA sequencing probes viral transcriptional activity at high depth, but rare transcripts or highly localized expression may escape detection in whole-insect datasets and contamination from content of the gut lumen may confound detection of very small amounts. Finally, Nanopore sequencing can reveal viral dsDNA molecules associated with viral replication. However, it cannot exhaustively capture every possible replication product and it also has a similar drawback of a possible slight contamination from gut lumen content. These caveats are not weaknesses specific to the present study; they are precisely why past debates on vector replication have often persisted when based on isolated markers.

The strength of the present work lies in the convergence of these independent approaches. Indeed, considering all results together, for FBNSV to replicate substantially in aphids, this replication would have to occur without detectable accumulation, without detectable replication protein, without detectable nuclear viral DNA, without detectable complementary-strand DNA, without detectable viral mRNAs and without detectable viral dsDNA. Such a scenario cannot be formally excluded in the absolute sense, but it would imply a process so weak, transient or spatially restricted that it is unlikely to contribute meaningfully to virus transmission.

In summary, our results provide experimental support for a view that has so far largely been assumed: FBNSV is transmitted by aphids in a genuinely circulative non-propagative manner. This conclusion does not require that replication be impossible under every conceivable condition, nor does it exclude biologically marginal events below current detection limits. More broadly, this work turns a reasonable but weakly documented assumption into a robust experimentally supported conclusion: if FBNSV replication occurs in aphids at all, it is unlikely to represent a significant step of the viral cycle.

## Supplementary material

10.1099/jgv.0.002354 Supplementary Material 1.

10.1099/jgv.0.002354 Supplementary Material 2.

## Abbreviations

AAP: acquisition access period
AMG: anterior midgut
BBTV: banana bunchy top virus
CP: coat protein
FBNSV: faba bean necrotic stunt virus
HCR-DNA-FISH: hybridization chain reaction fluorescence in situ hybridization
PFA: paraformaldehyde
PSG: principal salivary gland
qPCR: quantitative polymerase chain reaction
RT: room temperature
TYLCV: tomato yellow leaf curl virus
