# Supplementary material for: Converging lines of evidence fail to detect nanovirus replication in its insect vector
Source: J Gen Virol. 2026 Oct 27;107(10):002354. doi: 10.1099/jgv.0.002354 (PMC13713036; doi:10.1099/jgv.0.002354)
Supplement: Supplementary file 1 [file jgv-107-02354-s001.pdf]

Supplementary Material

Converging lines of evidence fail to detect nanovirus replication in its insect vector

Supplementary Table S1.

Sequences of the probes used for HCR-FISH

gene	accession	Adaptor used	Probe name	sequence	amplifier
FBNSV JKI-2000 segment U4	GQ150785.1	B1	N+1.1	gaggagggcagcaaacggaagttctcattaagaacggtaattata	B1-alexa 546nm
FBNSV JKI-2000 segment U4	GQ150785.1	B1	N+1.2	tttcaaatcacactgactatctaactagaagagtcttcctttacg	B1-alexa 546nm
FBNSV JKI-2000 segment U4	GQ150785.1	B1	N+2.1	gaggagggcagcaaacggaaaaatactgagactcgatgtcgagat	B1-alexa 546nm
FBNSV JKI-2000 segment U4	GQ150785.1	B1	N+2.2	tttcttctataatcttcatctttcttagaagagtcttcctttacg	B1-alexa 546nm
FBNSV JKI-2000 segment U4	GQ150785.1	B1	N+3.1	gaggagggcagcaaacggaaaatcattatgatacagataaccacc	B1-alexa 546nm
FBNSV JKI-2000 segment U4	GQ150785.1	B1	N+3.2	acgtcctctgaccttcgtaatagcctagaagagtcttcctttacg	B1-alexa 546nm
FBNSV JKI-2000 segment U4	GQ150785.1	B1	N+4.1	gaggagggcagcaaacggaaaccgtcgtcttccatcatagatatc	B1-alexa 546nm
FBNSV JKI-2000 segment U4	GQ150785.1	B1	N+4.2	tggtaaatatccaatcggtcgacaatagaagagtcttcctttacg	B1-alexa 546nm
FBNSV JKI-2000 segment U4	GQ150785.1	B2	N-2.1	cctcgtaaatcctcatcaaatgatagcatgaaagacaagctcaac	B2-alexa 647nm
FBNSV JKI-2000 segment U4	GQ150785.1	B2	N-2.2	aaatccacagcaagagtttatgtgtaaatcatccagtaaaccgcc	B2-alexa 647nm
FBNSV JKI-2000 segment U4	GQ150785.1	B2	N-3.1	cctcgtaaatcctcatcaaaagtgtgagcttcaatggtagtttct	B2-alexa 647nm
FBNSV JKI-2000 segment U4	GQ150785.1	B2	N-3.2	tcaagaaaggttctgcttgttagttaaatcatccagtaaaccgcc	B2-alexa 647nm
FBNSV JKI-2000 segment U4	GQ150785.1	B2	N-5.1	cctcgtaaatcctcatcaaaaattggtggttatctgtatcataat	B2-alexa 647nm
FBNSV JKI-2000 segment U4	GQ150785.1	B2	N-5.2	tgtttgtcgaccgattggatatttaaaatcatccagtaaaccgcc	B2-alexa 647nm
FBNSV JKI-2000 segment U4	GQ150785.1	B2	N-7.1	cctcgtaaatcctcatcaaaaacgggttagatagtcagtgtgatt	B2-alexa 647nm
FBNSV JKI-2000 segment U4	GQ150785.1	B2	N-7.2	aaatttataattaccgttcttaatgaaatcatccagtaaaccgcc	B2-alexa 647nm

Supplementary Statistical Analysis on RStudio.
Detailed statistical analyses corresponding to Fig. 1A and Fig. 1B.

1st Experiment (graph A, Figure 1):

```
library(readxl)
library(tidyverse)
library(rstatix)
library(ggpubr)
library(dplyr)
library(rcompanion)
library(lmtest)

data <- read_excel("Tableau Rep3.xlsx")
data <- data %>%
  mutate(
    Time = factor(Time),
    Plant = factor(Plant)
  )
str(data)
model <- aov(`Viral load` ~ Time * Plant, data = data)
residus <- residuals(model)
shapiro.test(residus)
str(data)
model <- aov(`Viral load` ~ Time * Plant, data = data)
residus <- residuals(model)
shapiro.test(residus)
        Shapiro-Wilk normality test

data:  residus
W = 0.73908, p-value = 4.471e-08

model <- lm(`Viral load` ~ Time * Plant, data = data)
bptest(model, studentize = TRUE)

      studentized Breusch-Pagan test
data:  model
BP = 17.289, df = 8, p-value = 0.02723

scheirerRayHare test

names(data)[names(data) == "Viral load"] <- "Viral_load"
scheirerRayHare(`Viral_load` ~ Time * Plant, data = data)
DV:  Viral_load
Observations:  50
D:  1
MS total:  212.5

           Df Sum Sq      H p.value
Time        2  140.3 0.6602 0.71886
Plant       2 1060.0 4.9881 0.08257
Time:Plant  4  380.6 1.7909 0.77415
Residuals  41 8770.2
```

2nd Experiment (graph B, Figure 1):

```
library(readxl)
library(tidyverse)
library(rstatix)
library(ggpubr)
library(dplyr)
library(rcompanion)
library(lmtest)

data <- read_excel("Tableau Rep3.xlsx")
data <- data %>%
  mutate(
    Time = factor(Time),
    Plant = factor(Plant)
  )
str(data)
model <- aov(`Viral load` ~ Time * Plant, data = data)
residus <- residuals(model)
shapiro.test(residus)

        Shapiro-Wilk normality test

data:  residus
W = 0.92906, p-value = 0.01677

model <- lm(`Viral load` ~ Time * Plant, data = data)
bptest(model, studentize = TRUE)

      studentized Breusch-Pagan test
data: model
BP = 20.777, df = 8, p-value = 0.007765
      scheirerRayHare test

names(data)[names(data) == "Viral load"] <- "Viral_load"
scheirerRayHare(`Viral_load` ~ Time * Plant, data = data)
DV:  Viral_load
Observations:  39
D:  1
MS total:  130

           Df Sum Sq      H p.value
Time        2  471.4 3.6262 0.16315
Plant       2  198.7 1.5281 0.46578
Time:Plant  4  432.6 3.3274 0.50461
Residuals  30 3958.9
```
